# The Wonder Dye: Uses and Implications of Indigocyanine Green in Various Surgeries

**DOI:** 10.7759/cureus.46722

**Published:** 2023-10-09

**Authors:** Nachiket P Rahate, Ankita Kapse, Prashant V Rahate, Sakshi P Nimbhorkar

**Affiliations:** 1 Surgery, Jawaharlal Nehru Medical College, Datta Meghe Institute of Higher Education and Research, Wardha, IND; 2 Medicine, Datta Meghe Medical College, Datta Meghe Institute of Higher Education and Research, Nagpur, IND; 3 Surgery, Seven Star Hospital, Nagpur, IND; 4 Medicine, Jawaharlal Nehru Medical College, Datta Meghe Institute of Higher Education and Research, Wardha, IND

**Keywords:** vascularity, scopes, tissue perfusion, near infrared imaging, indigocyanine green

## Abstract

Indigocyanine green (ICG) is a fluorophore dye that has been extensively used in recent modern times for bioimaging in numerous surgeries to aid in easier identification of occult and often tricky-to-find anatomical structures. Surgery becomes complex and challenging due to multiple anatomical anomalies, pathological fibrosis, obesity, or previous surgeries. To overcome these obstacles in surgery, the surgeon yearns to know the structures present beyond their white light vision so that while dissecting the organ, they can avoid injuring the critical systems in the vicinity of dissection. Near-infrared (NIR) imaging aids in visualising the tissues at depth/in the area of dissection, thereby preventing any possible surgical catastrophes due to them inadvertently damaging surrounding vital structures. Various advantages in surgeries like gastric sleeve surgery, lymph node and tumour detection, localisation of ureters and biliary tracts, and intraoperative tissue perfusion of flaps have been described in this study. This review article aims to compile a short list of utilities of ICG with NIR imaging in various surgical interventions. The merits and demerits of this imaging technique have been noted. The study points out the uses of ICG fluorescence imaging under different surgical fronts. This review article concludes by comparing the results of studies performed by various authors. Results have been compared to conventional surgical modalities.

## Introduction and background

In most scenarios, the surgeon has to rely solely on visual or palpatory cues to learn about the vascularity of the tissues and the filling of vital tubular structures. New intraoperative imaging systems using the near-infrared (NIR) part of the light spectrum (780 nm to 920 nm) are used intraoperatively to see the deeper tissues up to 10 mm from the surface [1]. NIR warrants an agent known as fluorophore such as indigocyanine green (ICG) dye, and an imaging apparatus (camera system and scope) that can both excite and detect the fluorophore (ICG) [2]. The significance of ICG dye with NIR imaging has not been well documented owing to its novelty in medical science. Aggressive clinical trials are needed to support and promote the use of this imaging modality in general surgical practices. This review article compares the results of a few cohort studies and randomised control trials and provides a considerable conclusion on the merits of ICG-NIR imaging.

## Review

Principle

**Figure 1:**
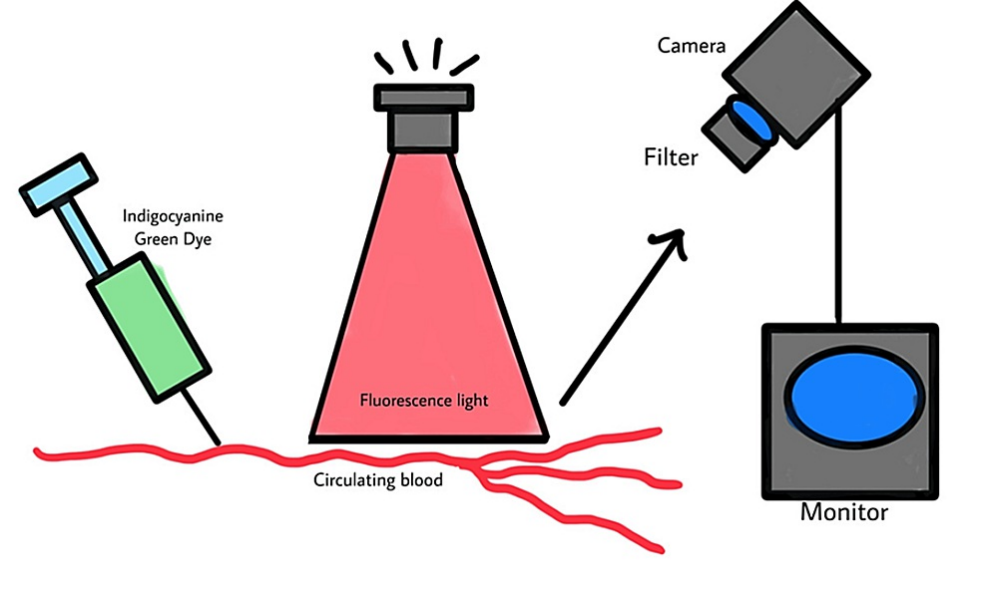
Action of Fluorescent Light on ICG Dye In Vivo Open access journal under a CC-BY license Contributed by Dr. Anna Duprée, published by De Gruyter [4]. ICG: indigocyanine green

The complete apparatus consists of ICG dye, a fluorescent light emitter, a camera that can sense excited ICG dye, and a monitor. Fluorescent light, a type of NIR, has a deeper penetration than normal white light. It can reach a depth of up to 10 mm from the superficial surface. Therefore, structures like blood vessels, tubular ducts, and visceral organs are susceptible to penetration. If an exciting agent like ICG dye is introduced into such tissues, it can get excited by the fluorescent light and radiate electromagnetic rays. These rays are picked up by a sensor, which processes the image. Structures and tissues with a higher gradient of oxygen perfusion emit more elevated amounts of electromagnetic rays, thus forming a brighter image on the monitor [3]. The mechanism has been visualised in Figure 1.

Method

ICG dye is injected intravenously 60-90 minutes before the surgery. For example, in cholecystectomy, the essential biliary anatomy and its variation, which is secluded from routine white light endoscopy, is easily visible by NIR. After initiating the surgery, the surgeon flips to NIR imaging mode because of which tissues and biliary structures at a depth of up to 10 mm are visible. This prevents any possible surgical catastrophes due to optical illusions caused by white light. Similarly, the same technology is used for various surgical procedures [5].

**Figure 2:**
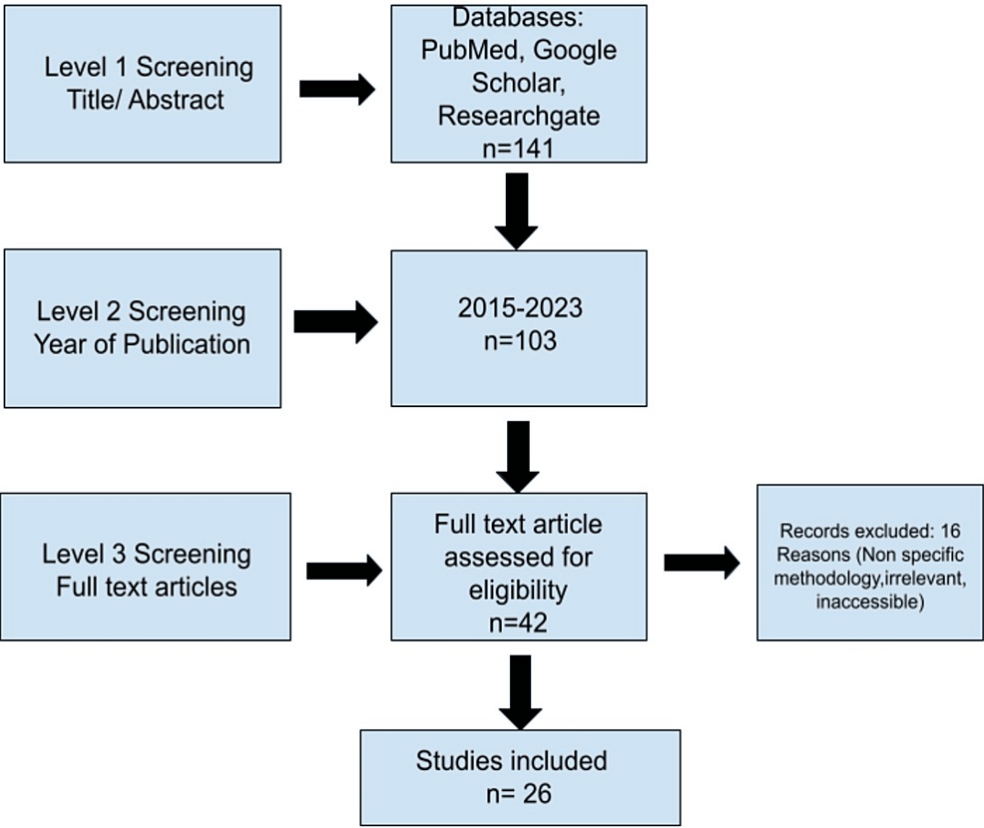
Flowchart Shows Identified Studies Included in this Review Article The flowchart is prepared by authors of this article.

The extensively reviewed databases were PubMed and ResearchGate. The following keywords: “ICG,” “NIR,” and “Vascularity” were searched from which 141 results were obtained. A filter was applied to include only the recent articles and studies ranging from years 2015 to 2023. The results were narrowed down to 106. A total of 26 articles were selected to be included in this review. More specific key terms were added as per the various surgical applications mentioned below. This review article uses references from a series of cohort studies and randomised control trials by Zhong et al. [6], Ambe et al. [7], Pavel et al. [8], Mizrahi et al. [9], Kalmar et al. [10] and White et al. [11] and their results have been tabulated [6-11]. All these study articles compare the use of conventional surgical methods and ICG dye with NIR imaging. Figure 2 shows the inclusion criteria for papers reviewed in this article.

Uses

Sentinel Lymph Node Mapping

Each organ has a sentinel lymph node where the lymphatics from that organ first drain. In the case of malignancy, for example, breast carcinoma, the lymphatics first drain into axillary lymph nodes. ICG dye is injected into the breast to identify the underlying lymph node affected. The dye takes 15 minutes to reach the lymph nodes [12,13]. The lymph node with the dye is excised, and an intraoperative frozen section study is performed. Further surgery depends on malignancy development in the excised lymph node. ICG and NIR imaging plays a crucial role in detecting lymph nodes for malignant transformation [14].

Lymphatic Structure Identification

Post-breast carcinoma excision, patients can develop chronic lymphatic oedema. For this, lympho-lymphatic anastomosis is performed. ICG dye is injected into the web spaces of fingers. ICG dye travels along the lymphatic channels in about 15 minutes and can easily be visualised. The site of obstruction is identified [15].

Tumour Detection

ICG dye is injected intravenously 3 to 7 days before assessment of the tumour. In this duration, ICG is excreted from the circulation but persists in tumour cells. This inherent affinity helps in identifying tumour cells and secondary malignancies [16]. Nagahara et al. suggest the preferential uptake of ICG dye by the tumour cells is due to the action of COX-2, nitric oxide synthesis activity, and aggressive neovascularisation [17-19].

Flap Vascularity in Plastic Surgery

A flap is a unit of tissue that is transferred from a donor site to a recipient site with an intact blood flow. A healthy flow of blood is necessary for a healthy anastomosis of tissue. ICG is injected intravenously, and NIR imaging is done. Within 15 seconds of dye administration, blood vessels can be visualised. Quantitative NIR imaging can differentiate a tissue into poorly, moderately, or healthy blood supply regions. Tissues with poor blood supply postoperatively must be operated on again to excise the ischemic area [20,21].

Anastomosis of Gastrointestinal Tract

In procedures for repair of ischemic gangrene or malignancies of the gastrointestinal tract (GIT), the affected segment is removed, and the proximal and distal ends of GIT are anastomosed together. Quantitative NIR imaging can be done within 15 seconds of ICG dye administration [22]. Quantitative NIR imaging can differentiate a tissue into poorly, moderately, or healthy blood supply regions. Tissues with poor blood supply postoperatively have to be operated on again to excise the ischaemic area [23].

Arterial Anatomy in Calot’s Triangle

One specific function of this modality is to identify any variants of cystic artery like the “Caterpillar hump [24].”If the patient has a caterpillar hump, the surgeon can cut the artery causing right hepatic ischemic necrosis. Thus, it becomes vital to identify this catastrophic variant and avoid the sequelae [25].

Detection of Vital Structures, That Is, Biliary Ducts and Ureters

**Figure 3:**
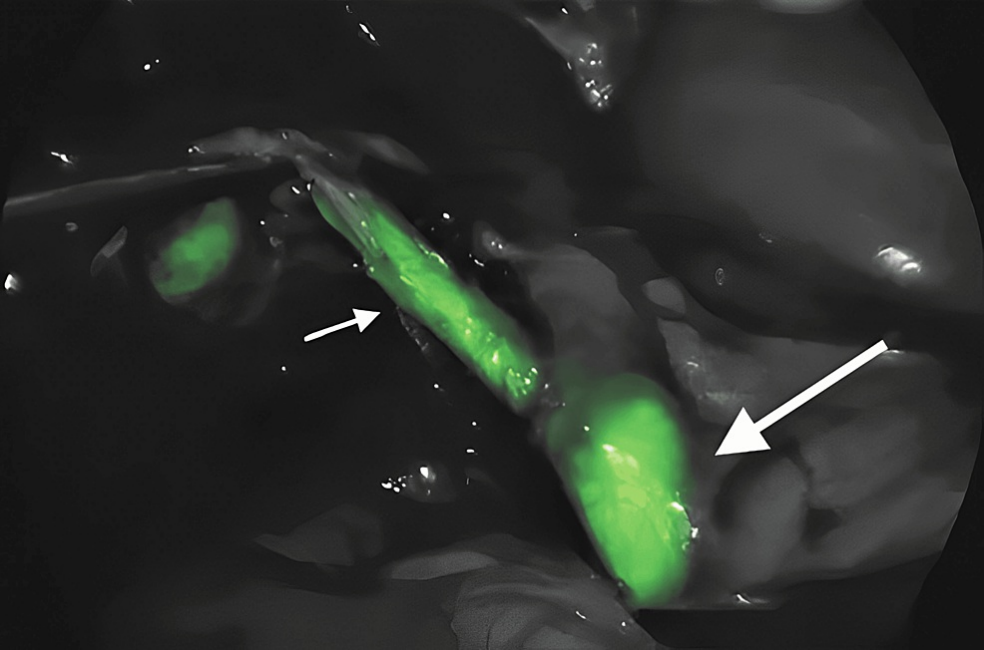
Laparoscopic View of Hepatobiliary System Illuminated by NIR Light The image is owned by the authors of this article. NIR: near-infrared

ICG should be injected intravenously 60 to 90 minutes prior to NIR imaging [7]. ICG is excreted through the liver and justifies its presence in the hepatobiliary system and ureters. Alternatively, a catheter with a piston of ICG dye can be inserted into the ureters to differentiate them from other structures during surgeries like hysterectomy [26,27]. As seen in Figure 3, the hepatobiliary structures appear green and stand out from surrounding tissues, making it easier for the surgeon to visualise them.

Gastric Sleeve Surgery

**Figure 4:**
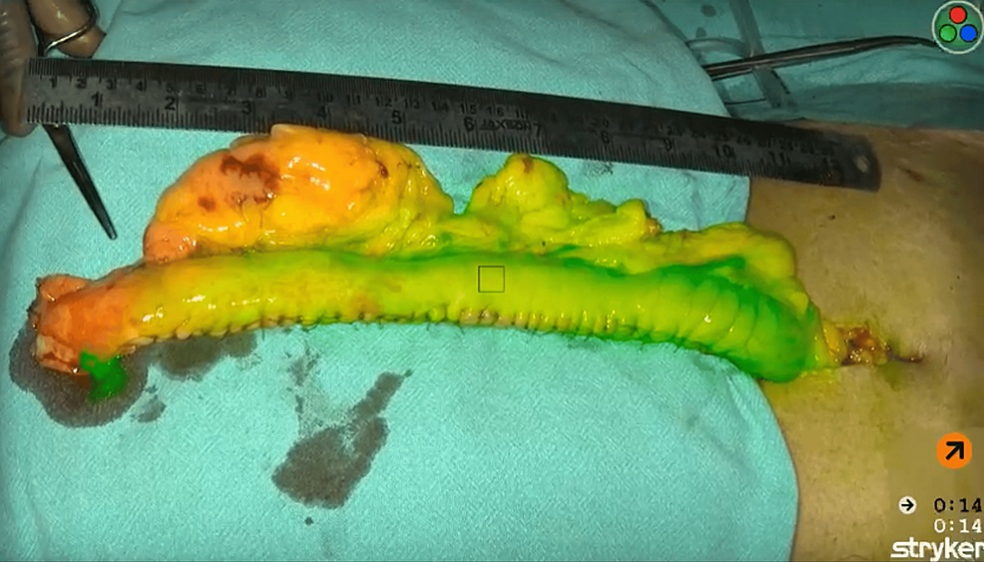
Viable Gastric Tissue under NIR Light in Green The image is owned by the authors of this article. NIR: near-infrared

During bariatric surgery, a gastric calibration tube is inserted into the stomach. This tube is filled with ICG dye. The tube is approximated along the lesser curvature of the stomach, and accordingly, the body of the stomach is resected. This leaves a sleeve parallel to the lesser curvature of the gut [28,29]. Figure 4 shows the ICG fluorescent dye in gastric tissue making it easier to visualise the viable tissue.

Parenchyma-Saving Adrenal Surgeries

In pathologies like pheochromocytoma, nodular involvement of adrenal glands is usually observed. However, present interventional modalities dictate resection of the entire adrenal gland. With experimental ICG administration and NIR imaging modalities, only affected nodules of the adrenal gland are removed, and the rest of the gland is preserved [30,31].

Drawbacks

ICG dye, a diagnostic aid, should be utterly harmless to the human body. Similar to an ideal dye, ICG is not toxic and is not metabolised by the liver. It has a rapid renal excretion and rarely causes any hypersensitivity reactions. On the other hand, it may contain fluorophore dot nanoparticles, which have been found not to get excreted by kidneys if it has a hydrodynamic diameter of 15 nm or more. This does not cause any short-term or acute pathologies, but for a long time, it has shown systemic damage in experimental animal subjects [32].

ICG dye shows a property of non-specific uptake by hepatic, splenic, and lymphatic tissues. These tissue-dye complexes get retained in the organs of the reticuloendothelial system, thereby acutely shortening its life span [33].

Some studies do not show the reduced advantages of ICG-NIR imaging over conventional surgical modalities. Ohdaira et al. report easier identification of sentinel lymph nodes in laparoscopy, but this did not affect the duration of the surgery or the postoperative recovery of the patient [34]. McGregor et al. report better visualisation of vascularity with ICG dye but limited to ventral cerebral tumours and deeply situated intramedullary tumours [13].

A cohort study by Li et al. suggests using ICG imaging for flap thickness of only 20 mm. Beyond 20 mm, the accuracy of identifying vascularity reduces significantly and the results obtained are not significant [35].

The study by Takami et al. concluded that ICG dye bound to human serum albumin (ICG-HSA) is more effective in identifying tumours than standalone ICG dye [18]. This may be attributed to the quicker lymphatic clearing of ICG dye. They also suggest the use of ICG dye for oesophageal cancers because of its poor lymphatic clearance. They advise using ICG-HSA complex dye for gastric, colorectal, bladder, cervical, prostatic, endometrial, and ovarian cancers. Unavailability of ICG-HSA thus plays an important factor in rapidly diagnosing these tumours.

Results

ICG dye with NIR imaging can be used in many surgical procedures. A few have been tabulated in the above table with their authors and remarks. Recommendations are based on the “p-value,” which reflects the probability of the results being accurate to the representation in an actual clinical setup. A p-value less than or equal to 0.1 is considered significant. Some studies recommend the use of ICG fluorescence imaging to reduce surgical error in identifying vital anatomical structures during surgical dissection [6,8-11]. Only a study by Ambe et al. showed that not enough studies were done to solidify ICG fluorescence imaging in laparoscopic cholecystectomy [7]. Still, they recommended using NIR over white light surgeries of the gallbladder. A survey by Li et al. showed the use of this modality for flap thickness less than 20 mm to have improved and healthy anastomosis [35]. Overall compliance and success rates in all studies suggest the adoption of ICG fluorescence imaging for surgeries of vital structures as seen in Table 1. Widespread adoption of the neo-modality can result in a drastic drop in intraoperative hazards. 

**Table 1:** Recommendations for ICG in Various Surgeries nm: nanometres; mm: millimetres; λ: wavelength; ICG: indigocyanine green

Authors	Materials used	Results	Comments
Zhong et al. (2021) [6]	SPY-Q by Novadaq, flexible 3D endoscope, λ=805 nm	Recommended (p=0.001)	100% of malignant lymph nodes were detected in all 385 subjects using ICG imaging.
Ambe et al. (2019) [7]	PinPoint by Novadaq, λ=805 nm	Should be considered (p=0.53)	Patients operated with ICG imaging had 2-5 minutes of shorter operating time and had an overall shorter duration of hospitalisation postoperatively.
Li et al. (2018) [35]	Real-time portable Novadaq camera, λ=835 nm	Recommended if flap thickness <20 mm	Improved visualisation of subcutaneous perforations up to 20 mm. Better for localisation of iatrogenic thrombus formation in flap surgeries.
Mizrahi et al. (2018) [9]	PinPoint camera, λ=805 nm	Recommended (p=0.49)	All 30 patients with ICG imaging had no anastomotic leaks compared to 6.67% of residual anastomotic leaks postoperatively in patients without ICG imaging.
Kalmar et al. (2020) [10]	Laparoscopy, flexible 3D endoscope, λ=820 nm	Recommended	100% sensitivity and 98.3% specificity in detecting gastric pouch abnormalities in bariatric surgery.
White et al. (2021) [11]	PinPoint camera, λ=835 nm	Recommended	94% of patients undergoing colorectal surgeries with ICG imaging had no iatrogenic urethral trauma. 0% adverse drug reactions after ICG administration.

Discussion

In most scenarios, the surgeon has to rely solely on visual or palpatory cues to learn about the vascularity of the tissues and the filling of vital tubular structures. ICG and NIR imaging modality (fluorescence imaging) has been proven to reduce the risk of various surgical complications such as anastomosis leaks, lymphatic obstructions, microscopic tumour cells by R2 resection, etc. This works on the principle that NIR light penetrates deeper tissue, excites the ICG dye and gets reflected onto an NIR sensor. Structures having affinity to the dye can be visualised. A study by Zhong et al. conducted a randomised clinical trial on 514 patients, out of which 385 subjects belonged to the ICG intervention group [6]. A significantly higher number of lymph nodes were identified in the ICG group. The diagnostic accuracy of diagnosing all gastric cancer and lymphadenitis cases reached 100%.

The study by Ambe et al. conducted a survey of 70 subjects undergoing laparoscopic cholecystectomy, out of which 29 and 41 subjects underwent cholecystectomy with and without ICG, respectively. Both groups were matched and had similar demographic details. The study showed that subjects undergoing cholecystectomy with ICG imaging had a shorter stay at the hospital, with their operation time being 2 to 5 minutes quicker when compared to cholecystectomy without ICG imaging.

In the prestigious study by Li et al., it was concluded that ICG fluorescence imaging significantly aided the detection of microvessels and subcutaneous perforations in tissues up to 20 mm [35]. This enabled the surgeons to choose viable and healthy tissues with adequate anastomosis. It also helped in detecting any newly formed iatrogenic thromboses in the microvessels.

Out of the 60 patients in the study conducted by Mizrahi et al., 30 patients underwent repair of the anastomosis with the help of ICG imaging [9]. No patients had clinically and radiologically detected an anastomotic leak, compared to two patients operated without ICG fluorescence imaging.

The paper by Kalmar et al. studies 196 patients undergoing bariatric surgery, out of which 59 patients were operated on with the help of ICG imaging [10]. It had 100% sensitivity with 98.3% specificity. The study concludes that the given modality has a comparable specificity to conventional gastroscopy.

In the study by White et al., 94% of patients underwent colorectal surgeries aided by ICG-NIR imaging in which no patients acquired an iatrogenic intraoperative ureteral trauma [11]. This was accompanied by 0% adverse reactions and detrimental sequelae to intravenous ICG dye administration. The study concluded that ICG dye administration was safe and an effective modality for visualising the ureter and other vital organs.

ICG imaging is undeniably a blessing in disguise for surgeons, but it is poorly understood. The lack of expertise and availability of the required modalities can be a significant obstruction in the propagation of the use of this imaging. A unanimous consensus must be drawn about the specificities of the methodology.

## Conclusions

ICG fluorescence (NIR) imaging has been a boon for surgeons worldwide. It is a safe and effective interventional modality for preventing iatrogenic trauma to vital structures. Broad implications and uses have been identified in numerous surgical interventions that reduce the risk of the development of complications of surgeries usually done under white light. Results from various studies indicate a comprehensive promotion and use of ICG fluorescence (NIR) imaging modality. Due to a lack of knowledge, good service has not been achieved. This has resulted in non-favourable results in some of the existing trials. Some studies above show positive results, but the advantages are not financially and medically justified. Therefore, further studies are required to strengthen the advocacy for its use. Major demographic, practical, and expertise deficits are present in the current understanding of ICG-NIR imaging.

Extensive studies and profound knowledge can help catapult the uses of it. ICG is just one of the many fluorophores that are being used in medical sciences for diagnostic purposes. Many more dyes and surgical inks have been employed in various techniques for intraoperative visualisation of tissues. The ICG dye has proven to be a game-changer in surgeries in the matter of just a few years. Use of ICG and other dyes can help prevent innumerable intraoperative complications.
